# Cardiovascular imaging 2011 in the International Journal of Cardiovascular Imaging

**DOI:** 10.1007/s10554-012-0040-6

**Published:** 2012-04-05

**Authors:** Ricardo A. Costa, Hiram G. Bezerra, Johan H. C. Reiber, Frank J. Rybicki, Paul Schoenhagen, Arthur A. Stillman, Johan De Sutter, Nico R. L. Van de Veire, Ernst E. van der Wall

**Affiliations:** 1 Instituto Dante Pazzanese de Cardiologia, São Paulo, Brazil; 2 University Hospitals Case Medical Center, Cleveland, OH USA; 3 Department of Cardiology, Leiden University Medical Center, Albinusdreef 2, 2333 ZA Leiden, The Netherlands; 4 Harvard Medical School, Boston, MA USA; 5 The Cleveland Clinic Foundation, Cleveland, OH USA; 6 Emory University Hospital, Atlanta, GA USA; 7 AZ Maria Middelares Gent and Free University Brussels, Brussels, Belgium; 8 Division of Image Processing (LKEB), Department of Radiology, Leiden University Medical Center, Albinusdreef 2, 2333 ZA Leiden, The Netherlands

## Introduction

Similarly as we did in 2010, please, find below our annual overview of the published papers in the International Journal of Cardiovascular Imaging in the year 2011. We believe that we have received again very interesting papers over the last year, which we have subdivided over the well-known areas, i.e. X-ray angiography, intravascular imaging, echocardiography, nuclear cardiology, magnetic resonance imaging, and computed tomography. In 2011 we published two Topical issues, one on QCA, IVUS and OCT in interventional cardiology (Guest Editors HG Bezerra, RA Costa and HM Garcia-Garcia, Vol. 27, no. 2), and one on Transcatheter Valvular Interventions (Guest Editors JJ Bax and P Schoenhagen, Vol. 27, no. 8). In addition, the Asian Society of Cardiovascular Imaging published two ASCI Special issues with Adjunct Editor YH Choe and Guest Editors BW Choi, H Sakuma and J Lee, the first one being Vol. 27, no. 5 the second one Vol. 27, Suppl 1.

## X-ray angiography

The second volume in 2011 was a Topical issue on QCA, IVUS and OCT in interventional cardiology. In the QCA section a number of important developments in this field were presented, starting with an overview on angiographic imaging and scoring techniques by Ng et al. [1] entitled “Novel QCA methodologies and angiographic scores”. A total of 10 scoring systems are described. They also conclude that QCA will continue to evolve as 3D coronary angiographic techniques become incorporated into routine angiography to provide more detailed descriptions of complex lesions, such as bifurcations, eccentric lesions and chronic total occlusions. Furthermore, scoring systems which include both clinical and angiographic characteristics are still under development and will require validation. In the manuscript by Tuinenburg et al. [2] the basic principles as well as validation results of two models for coronary bifurcations are presented. These two models, the T- and Y-shape model, are selected depending on the anatomy of the bifurcation. They conclude that the accuracy, precision and applicability of these new bifurcation analyses are in agreement with the general guidelines that were set many years ago for conventional QCA-analyses. Holm et al. [3] have applied the software described in the previous manuscript in pre-PCI, post-PCI and 8-months FU in 957 patients in the first three Nordic Bifurcation studies. They indicate in their manuscript that an elaborate standard operating procedure (SOP) is still necessary and essential for standardized high quality QCA analyses of bifurcation lesions. They have contributed much to these standardizations, which are essential in any research environment. Based on their extensive experience with various dedicated bifurcation devices, Collet et al. [4] have reviewed the QCA results of a number of such devices using the new QCA segmental analysis. They divide the bifurcation stents into four categories: (1) dedicated parent vessel (PV) devices; (2) dedicated PV with side-branch (SB) access port; (3) dedicated SB devices; and (4) dedicated PV plus SB devices. Among others, they conclude that the mean carina angle may have prognostic value and device-specific implications.

Costa et al. [5] reported about the bifurcation lesion morphology and the intravascular ultrasound assessment. It is their view that the bifurcation lesion anatomy and morphology is critical for clinical decision making, and a key factor for successful bifurcation PCI. Among others, the following factors need to be considered: (1) optimal angiographic viewing; (2) current lesion classification may not be sufficient; (3) dedicated bifurcation QCA is needed; (4) IVUS assessment of bifurcation lesions pre-procedure provide valuable information; and (5) at post-procedure, IVUS provides valuable and prognostic information about the stent apposition. The last manuscript on X-ray imaging concerns the work by Tu et al. [6] about the fusion of 3D QCA and IVUS/OCT. They have developed and validated a novel fusion technique based on the 3D QCA from either mono- or biplane angiographic views and the registration with IVUS or OCT pullback sequences. This new and fast approach allows the interventional cardiologist with detailed information about the vessel and plaque size at every position along the segment of interest, and avoids all the guess work about the cross-sectional IVUS or OCT data and the actual position in the coronary artery.

Al-Hay et al. describe the efficacy and safety of the Amplatzer septal device for percutaneous occlusion of Fontan fenestration in [7] in a retrospective review of 26 consecutive patients, who underwent transcatheter closure of Fontan fenestration in a tertiary cardiac center in Kuwait. They reported a 100 % occlusion rate and that no complications or device failures were seen during follow-up. Finally, Hsieh et al. [8] described the role of three-dimensional rotational venography supplementary to two-dimensional (2D) digital subtraction venography (DSV) in the evaluation of the left iliac vein in patients with chronic lower limb edema in a group of 34 patients. They concluded that in patients with negative 2D images, additional 3D RV leads to higher diagnostic sensitivity, thereby providing a powerful tool for planning surgery and endovascular treatment.

## Intravascular imaging

Intravascular ultrasound (IVUS) and Intravascular optical coherence tomography (IOCT) are two catheter-based intravascular imaging technologies. IVUS has played a pivotal role in the evolution of interventional cardiology, allowing a better comprehension of coronary artery disease (CAD) as well as technique and device improvements. IOCT, a near-infrared light-based technology, was more recently introduced and has 10 times the spatial resolution of IVUS and a very high contrast between lumen and vessel wall contour, since images are obtained in a virtual blood free environment. These improvements enable for the first time in vivo direct characterization of metrics like fibrous cap quantification and stent coverage. IVUS radio frequency derived technology facilitates plaque composition interpretation and quantification, which consequently can be applied in a wide range of research activities. We are presenting here selected publications that exemplify not only research potential, but also clinical applications of these technologies.

### Research applications; device evaluation

The International Journal of Cardiovascular Imaging received in the year 2011 several interesting contributions focused on the evaluation of vascular response following drug-eluting stent implantation as well as scaffold assessment. Chamié et al. [9] evaluated, longitudinally, 38 individuals at 8- and 20-months follow-up following durable polymer sirolimus-eluting stent implantation to explore the presence of late “catch-up”. The authors demonstrated by means of greyscale IVUS a slight, non-significant, increase in neointimal hyperplasia between the time points, which was interestingly restricted to the distal part of the stent. Brugaletta et al. [10] evaluated the temporal changes of atherosclerotic plaque 6 months after the implantation of everolimus-eluting bioresorbable vascular scaffolds (BVS) using VH-IVUS. An increase in plaque and external elastic membrane (EEM) areas coupled with an increase in necrotic core and fibrotic tissue were demonstrated behind the stent struts. Similar assessment of BVS using VH-IVUS at 6 months and 2-year follow-up was performed in 30 patients by Shin et al. [11] using a different methodology of analysis (i.e., Shin’s method) in which contours are drawn around the IVUS catheter (instead of delineating the lumen) and the vessel. The authors demonstrated a reduction of 20.5 % in necrotic core and 27.2 % in dense calcium areas at 2 years. Devices companies have recently adopted IOCT derived metrics as surrogated endpoint for device safety, in particular the 15 μm axial resolution of the method enables for the first time assessment of stent coverage which has been postulated to be a potential surrogate for stent thrombosis. Tahara et al. [12] carefully reviewed preclinical and clinical investigations that used stent strut coverage, as determined by OCT, as a surrogate safety endpoint in the evaluation of drug-eluting stents. The authors highlighted that although OCT surrogate endpoints have been used increasingly, it is still to be determined whether OCT can predict clinical outcomes, such as late stent thrombosis. As one example of having strut coverage as primary endpoint the paper by Kim et al. [13] used OCT surrogate endpoints to evaluate vascular response after the implantation of durable polymer paclitaxel-(PES) and sirolimus-eluting stents (SES) in long-term follow-up (≥2 years). In comparison with 9-months follow-up, while PES showed similar rates of uncovered and malapposed struts, SES demonstrated a trend towards a reduction in these percentages at very-late follow-up, more interestingly complete coverage is unusual even at very late follow-up. Mehanna et al. [14] performed a comprehensive review on the offline assessment of coronary stents by OCT. Qualitative and quantitative parameters regarding stent-vessel interactions were discussed extensively and serves as a good reference for researches considering IOCT as the imaging modality of choice for stent evaluation.

### Research applications; plaque characterization

Garcìa-Garcìa et al. [15] performed a comprehensive review on tissue characterization based on IVUS-derived technologies, namely virtual histology IVUS (VH-IVUS), iMap-IVUS, Integrated Backscatter IVUS, and Automated Differential Echogenicity. The strengths and weaknesses of each method were discussed, and the authors concluded that there is still an extensive debate on how these methods relate to each other and accuracy versus histopathology. Shin et al. [16] sought to correlate percent plaque components derived by plaque- and vessel-based measurements using VH-IVUS in 206 patients. The authors found that plaque volume and vessel volume were well correlated irrespective of plaque burden and clinical states and the percent plaque components volumes calculated by plaque volume correlated well to those calculated by vessel volume. Zhang et al. [17] used a preclinical pig model to validate IVUS elastography against gold standard histology. The authors were able to characterize fibro-fatty and fibrotic plaque, as well as the presence of macrophages with high sensitivity. Kubo et al. [18] showed important aspects of atherosclerotic plaque as well as thrombus characterization by IOCT. The capability of measuring fibrous cap thickness overlying a lipid plaque coupled with good accuracy in the identification of inflammation and thrombus, as shown by the comparison with standard histology, demonstrate that OCT might play an important role in the identification of plaque vulnerability. Furthermore, the authors highlighted that OCT, differently from IVUS, enables accurate quantification of calcium.

### Clinical applications

The assessment of coronary bifurcation lesions by IVUS was extensively reviewed by Costa et al. [5]; practical aspects to be considered on a daily basis in the catheterization laboratory, such as pre-stent evaluation of vessel size, plaque morphology and its distribution, as well as post-procedure assessment of stent expansion and apposition were highlighted. Shin et al. [19] applied the methodology (i.e., Shin’s method) using VH-IVUS to identify dense calcium volume and percent necrotic core to EEM as independent predictors of CK-MB elevation post successful, elective stent implantation. Stefano et al. [20] published the first comparison of OCT and fractional flow reserve (FFR) for the assessment of intermediate coronary artery stenosis. The authors demonstrated a clear complementary role between physiological and anatomical evaluations to guide decision making in complex clinical scenarios. OCT was valuable to rule out plaque rupture, erosion, and thrombosis in acute coronary syndrome patients; moreover, it helped guiding PCI strategy in tandem lesions with FFR < 0.80. This paper is particularly contemporary considering that two IOCT manufactures (St. Jude Medical, Inc and Volcano Therapeutics, Inc) detain both technologies. Also, recently St. Jude Medical launched an integrated IOCT/FFR system (ILUMIEN™). Consequently we can anticipate for the year 2012 an increased number of publications and research involving concomitant usage of both IOCT and FFR technologies.

Finally, a special highlight to the paper by Tu et al. [6] who performed a robust validation for the co-registration of three-dimensional quantitative coronary angiography (3-D QCA) with IVUS and OCT using in vitro and in vivo data. This provided information is a very important step for the complete fusion of 3-D QCA and IVUS/OCT. The authors pointed out that the information provided by this system combination could be used to correct for the error in quantifying plaque volume introduced by vessel tortuosity. Therefore, the information provided makes this combined system an important tool in the catheterization laboratory. As important as the technical aspects highlighted on this paper is the practical aspect of co-registration between different imaging modalities. The very heterogeneous adoption of intravascular methods, around the world, in part can be explained by the lack of integration between angiography and these methods. The paper by Tu et al. [6] is bringing to the surface an old topic, co-registration between imaging modalities. We hope to hear more on this topic in the near future since co-registration methods have the potential to effectively positively impact the adoption of intravascular imaging in daily practice.

In summary intravascular methods have been applied in a wide range of indications from plaque characterization to device evaluation. Most of the papers published in 2011 in the International Journal of Cardiovascular Imaging are focused in a variety of research applications of these methods. Considering the robustness achieved by these methods and continuous evolution we expect, for the upcoming years, more papers with emphasis in clinical applications of these methods in particular their potential impact in patient care.

## Echocardiography

Just like last year, The International Journal of Cardiovascular Imaging received in 2011 several interesting contributions dedicated to conventional echocardiography but also to emerging ultrasound techniques including intravascular ultrasound, stress echocardiography, tissue Doppler imaging, strain analysis and three-dimensional echocardiography [21].

### Conventional echocardiography

In this era of modern technology, we sometimes forget the value of ‘basic’ techniques applied in a continent with a huge cardiovascular morbidity and mortality: Africa. Mocumbi et al. [22] discussed the potential of echocardiography to foster research into cardiovascular neglected diseases in Africa. The challenges faced by the African research community are described in detail in this report. One of these neglected diseases is endomyocardial fibrosis (EMF), a cardiomyopathy with high prevalence in Sub-Saharan Africa with unclear etiology, pathogenesis and natural history. Mocumbi et al. designed a study aiming at assessing its accuracy in defining EMF structural abnormalities pre-operatively, and describe pathological findings through detailed intraoperative examination and evaluation of histopathological changes in tissue obtained from excisional biopsies. Severe EMF assessed by echocardiography was associated with intense endocardial fibrosis on histology [23]. Structural abnormalities of chronic severe EMF are accurately diagnosed by transthoracic echocardiography, allowing this non-invasive technique to be used as the gold standard for diagnosis and surgical management of chronic EMF in endemic areas. The pivotal role of conventional echocardiography in the diagnosis, understanding of pathophysiology, assessment of disease severity and patient monitoring in pregnant women with unoperated and post-operative congenital disease was discussed extensively in a review by Vitarelli and Capotosto [24].

### Dobutamine stress echocardiography

The prognostic value of dobutamine stress echocardiography (DSE) for risk stratification of patients aged ≥80 years is not clearly defined. Innocenti et al. [25] obtained follow-up of 3 ± 2 years for major cardiac events and all-cause mortality in 227 patients, age ≥ 80 years, who underwent DSE for known or suspected coronary artery disease. DSE showed a significant prognostic value in octogenarians, both for all-cause mortality and major cardiac events.

### Tissue Doppler imaging and two-dimensional strain/strain rate imaging

Sachdeva et al. [26] evaluated the association of tissue Doppler imaging and catheter-derived measures with rejection in pediatric heart transplant recipients and to determine any correlation between tissue Doppler imaging and catheter-derived measurements. Sixty echocardiograms were prospectively performed in 37 pediatric heart transplant recipients at the time of surveillance cardiac biopsy. During right-heart cardiac catheterization, sequential pressures of the right heart and pulmonary capillary wedge pressures were measured. Some tissue Doppler imaging derived measures were altered during rejection, but were not clinically useful predictors of rejection. Catheter-derived measures were not significantly altered during rejection and did not correlate with tissue Doppler imaging derived measures. The authors concluded that none of these measures could replace the current practice of performing cardiac biopsy for surveillance of rejection. Butz et al. [27] hypothesized that two-dimensional strain might be useful as additional tool in differentiating between pathologic and physiologic hypertrophy in top-level athletes. They studied 53 subjects, 15 patients with hypertrophic cardiomyopathy, 20 competitive top-level athletes, and a control group of 18 sedentary normal subjects. All components of strain were significantly reduced in patients with hypertrophic cardiomyopathy (global longitudinal strain: −8.1 ± 3.8 %; *P* < 0.001) when compared with athletes (−15.2 ± 3.6 %) and control subjects (−16.0 ± 2.8 %). This technique could offer a unique approach to quantify global as well as regional systolic dysfunction, and might be used as new additional tool for the differentiation between physiologic and pathologic left ventricular hypertrophy. Simsek et al. [28] evaluated left ventricular tissue Doppler imaging and strain/strain rate imaging properties in athletes and sedentary controls. This study demonstrated that left ventricular strain/strain rate imaging was higher in athletes than in healthy subjects. In addition to traditional echocardiographic parameters, strain/strain rate imaging could be utilized as a useful echocardiographic method for cardiac functions of athletes. Normal values: Reckefuss et al. [29] measured longitudinal strain (in three apical views) and radial strain (in short-axis view) in 144 healthy individuals ranging between 17 and 80 years. Their results may serve as a reference for comparison with different disease entities. Faber et al. [30] measured peak systolic longitudinal strain of the basal septum and the opposite lateral wall in 88 consecutive patients with obstructive hypertrophic cardiomyopathy who underwent a septal ablation procedure. At 12 months follow-up, peak systolic longitudinal strain improved significantly in the opposite lateral wall as a result of afterload reduction by elimination of the outflow gradient.

### Three-dimensional echocardiography

Chen et al. [31] evaluated the importance of factors affecting the efficiency of real time three-dimensional echocardiography image display quality and established an optimized method for real time three-dimensional echocardiography examination and image processing in children. Their study could help to design a multi-parameter presetting of ultrasound systems. Wu et al. [32] used three-dimensional imaging and strain imaging to study the relationship of myocardial segmental mechanics and regional volume change. This elegant mechanistic study provides insight into intraventricular dyssynchrony in patients with left ventricular systolic dysfunction.

### Intravascular ultrasound

In a special focus issue, the role of intravascular ultrasound (IVUS) was reviewed by several authors. Costa et al. [5] discussed the value of IVUS in the evaluation of bifurcation lesions. IVUS provides valuable information regarding vessel size and plaque morphology and distribution that may help select treatment strategy. At post-procedure, IVUS evaluates stent expansion, what may impact long-term outcomes. Tu et al. [6] reported on a new registration approach for the integration/combination of X-ray angiographic and IVUS/OCT imaging. This provides the interventionalist with detailed information about vessel size and plaque size at every position along the vessel of interest. Three review articles demonstrate the broad spectrum of current IVUS applications and underline the significant role of IVUS, highlighting the complementary roles of IVUS and OCT [15, 33, 34].

Finally, the last issue of the Journal was dedicated to emerging technology: *transcathether valvular interventions*. Multi-modality imaging is of paramount importance during the selection process of patients, the implantation of the valve and during follow-up. The role of conventional echocardiography but also the role of three-dimensional transoesophageal echocardiography during the implantation of the MitraClip device or during the implantation of percutaneous aortic valves was highlighted by Maisano and Siegel [35, 36].

## Nuclear cardiology

In 2011 several excellent papers were published in the field of nuclear cardiology. In this overview we highlight a selection of these papers.

### Technological advances

#### Stem cell therapy in heart failure

Cell therapy is an interesting therapeutic option in heart failure patients as it potentially improves contractility and restores regional ventricular function. However cell therapy remains complex and, next to determining the best cell type, the optimal delivery strategy, the bio-distribution and the survival of implanted stem cells after transplantation needs to be elucidated. Van der Spoel et al. [37] presented this year a comprehensive review in the journal on the various cell tracking techniques to observe the fate and the distribution of transplanted cells using non-invasive imaging techniques. These comments are highly important in view of currently on-going experimental and clinical studies.

#### Impact of CT attenuation correction on the viability patterns assessed by tetrofosmin SPECT/FDG PET

SPECT myocardial perfusion imaging is commonly used for comprehensive interpretation of metabolic PET FDG imaging in ischemic dysfunctional myocardium. Nkoulou et al. [38] evaluated the difference in scan interpretation introduced by CT attenuation correction (CTAC) of SPECT MPI in 46 patients undergoing viability characterization by Technetium SPECT MPI/PET FDG. Applying CTAC introduced a different reference segment for the normalization of PET FDG study in 57 % of cases. As a result, 25 % of segments originally classified as scar were reclassified and the number of normal segments increased by 20 %. Introducing CTAC decreased the number of patients with possible indication for revascularization by 54 %. Therefore, different interpretation of myocardial viability can be observed when using CTAC instead of using SPECT MPI without attenuation correction and this may result in clearly different clinical decision making.

#### Evaluation of left ventricular volumes in patients with idiopathic dilated cardiomyopathy

Sipola et al. [39] prospectively compared left ventricular (LV) volumes and LVEF both by 99 m Tc-tetrofosmin gated SPECT and MRI within a 3 h interval in 21 patients with idiopathic dilated cardiomyopathy. Excellent correlations between both methods were observed for LV volumes, but correlations were significantly weaker for LVEF (r = 0.323, *P* = 0.08). LV volumes were also significantly lower when evaluated by gated SPECT as compared to MRI. In 4 patients (21 %) the LVEDV index was considered normal by gated SPECT and increased by MRI if MRI-derived normal values were used. No differences in LVEF were found between gated SPECT and MRI when MRI-derived LVEF was below 40 %. However, gated SPECT showed lower LVEF when MRI-derived LVEF was over 40 % (33 vs. 50 %, *P* < 0.05).

The authors concluded that LV volumes are lower by GSPECT as compared to MRI and that no direct comparisons can be made between methods in follow-up studies. The authors also suggest that abnormal gated SPECT results should be confirmed by another imaging modality, such as MRI, if these findings have therapeutic consequences. These findings were also discussed in an editorial comment by van der Wall et al. [40]. They point out that the underestimation of LV volumes and LVEF in some patients might be due to the inclusion of the outflow tract area by MRI, which is not part of LV volume acquisition with gated SPECT because of low counts in this area. This holds in particular for dilated left ventricles as the basal left ventricle constitutes a higher absolute volume than in patients without left ventricular dilatation.

### Safety aspects of adenosine SPECT

In patients after cardiac transplantation, denervation super-sensitivity to adenosine is well described, particularly early after transplant. Al-Mallah et al. [41] now reported on the safety and hemodynamic effects of adenosine SPECT in a large series (n = 102) of patients late after transplantation (average 8 years). In comparison to an age-gender matched control group, cardiac transplant patients experienced a higher incidence of sinus pause (4.9 vs. 0 %), second degree AV block (11.8 vs. 4.9 %) and third degree AV block (2.9 vs. 0 %) (all *P* < 0.05). However, only 1.9 % of adenosine SPECT studies were terminated due to brady-arrhythmias with 1 patient requiring aminophylline. These findings indicate that denervation super-sensitivity can persist late after cardiac transplantation. As a result, adenosine induced brady-arrhythmias may occur more frequently than in non-transplant patients, but these arrhythmias are generally short-lasting and benign.

### Evaluation of coronary artery disease and myocardial ischemia in atrial fibrillation patients

Atrial fibrillation (AF) has been linked to the presence of underlying coronary artery disease (CAD). Nucifora et al. [42] evaluated 87 patients with paroxysmal or persistent AF with MSCT coronary angiography and stress testing (exercise ECG or myocardial perfusion imaging) and compared their findings to 122 age and gender, symptomatic status and pre-test likelihood matched patients without a history of AF. Obstructive CAD, as defined by MSCT, was present in 40 % of AF patients and in 25 % of non-AF patients (*P* < 0.05). However, in these patients with documented obstructive CAD, the prevalence of a positive stress test was comparable between both groups (49 vs. 48 %, *P* = ns). From these data it can be concluded that the higher burden of CAD observed in AF patients is not associated with a higher burden of myocardial ischemia.

### Reverse remodeling after revascularization in ischemic cardiomyopathy

Patients with ischemic cardiomyopathy can show varying degrees of LV remodeling after cardiac surgical revascularization. Skala et al. [43] directly compared myocardial perfusion SPECT and cardiac MRI to predict improvement of LVEF 24 months after CABG. They prospectively enrolled 53 patients with stable ischemic heart disease and mean LVEF of 35 %, of which 37 patients underwent CABG. A fixed perfusion defect on SPECT <16.5 % of LV predicted reverse remodeling (defined as a minimal increase of 5 % of LVEF after 24 months follow-up) with a sensitivity of 64 % and a specificity of 69 % (AUC 0.64). However, a preoperative number of ≤5 segments with delayed enhancement/wall thickness ratio of ≥50 % obtained by MRI was found to be better predictor of LV reverse remodeling (sensitivity 86 %, specificity 75 %, AUC 0.81). No other MRI of SPECT parameter predicted LVEF improvement at 24 months after CABG. These data suggest that delayed enhancement cardiac MRI is a better predictor of reverse remodeling after CABG as compared to myocardial perfusion SPECT. The study however, had a small sample size and lacked clinical outcome data regarding morbidity and mortality.

## Magnetic resonance imaging

There were a number of papers relating to the evaluation of patients with ischemic heart disease by cardiac magnetic resonance (CMR) in 2011. Lubbers et al. [44] examined the inter-observer variability of stress only adenosine first pass myocardial perfusion imaging and found that performance is experience related, but the systemic use of reading criteria significantly increased performance for less experienced observers. First-pass MR perfusion imaging with adenosine was compared to high-dose dobutamine/atropine stress in a series of 41 patients and found comparable high sensitivity and specificity for stenosis detection on a per patient basis [45]. Combining coronary calcium scoring was found to significantly improve the diagnostic accuracy of first pass myocardial perfusion and delayed enhanced imaging with CMR [46]. Lubbers et al. [47] examined the performance of adenosine stress only perfusion in patients without history of myocardial infarction in a series of 139 patients and found a very high negative predictive value for major adverse coronary events (MACE). The prognostic value of combined MR perfusion imaging and late gadolinium enhancement was studied in patients with known or suspected coronary disease [48]. Multivariate analysis showed that myocardial ischemia was the strongest predictor for hard cardiac events and MACE. The cost effectiveness of adenosine MR was studied in patients with suspected coronary artery disease [49]. It was found that CMR significantly reduces the utilization of cardiac catheterization and reduced per patient cost by a mean of € 90. Stensaeth et al. [50] reported the clinical characteristics of patients with suspected ST-elevation myocardial infarction and normal coronary arteries. Patients with monomorphic ventricular tachycardia (VT) were found to have larger scar volumes than patients with polymorphic VT, suggesting that CMR may be used for better risk stratification in patients with ischemic cardiomyopathy undergoing implantable cardioverter-defibrillator therapy [51]. Kino et al. [52] compared a navigator free breathing 3D phase sensitive inversion recovery (PSIR) TurboFLASH sequence to an established 2D PSIR method and found more hyper-enhanced scars and larger scar volume with the 3D technique suggesting potential advantages for scar assessment.

Delayed contrast enhanced CMR was found to be useful for differentiating cardiac tumors from thrombi in a small series of stroke patients [53]. Late gadolinium enhancement and T2-weighted CMR was found to be useful for differentiating acute from chronic myocardial injury in myocarditis [54]. Cheng et al. [55] reported the CMR imaging characteristics of isolated left ventricular noncompaction and found diagnostic accuracy when the noncompacted/compacted ratio was >2.5. Kim et al. [56] reported an interesting case of midwall myocardial fat deposition in a patient with dilated cardiomyopathy seen by both CT and MRI that may be related to apoptosis.

Fernández-Golfín et al. [57] found significant differences in left ventricular volume and function in patients with functional mitral regurgitation. Moreover, this group found significant differences in various mitral valve parameters in patients with and without significant mitral regurgitation, and these parameters differ between ischemic versus non-ischemic left ventricular dysfunction groups. In another functional study, Holloway et al. [58] found that visual inspection underestimated quantitative assessment of left ventricular ejection fraction.

Contrast enhanced whole-heart MRA at 3 T was found to be useful for assessing coronary venous anatomy [59]. Normal values for the geometry and dimensions of the pulmonary artery bifurcation were established by contrast-enhanced MRA [60]. Prompona et al. [61] found MRI to be useful for evaluating sinus venosus atrial septal defects and anomalous pulmonary venous drainage.

Metz et al. [62] found that ultra-small superparamagnetic iron oxide particles may be used to assess vascularity and macrophage content in atherosclerotic carotid plaques in a small series of patients. Wang et al. [63] found that carotid plaques in patients with acute coronary syndrome have a higher prevalence of fibrous cap rupture than patients with stable coronary artery disease. Sadat et al. [64] performed finite element stress simulations of carotid atherosclerotic plaques studied by MRI and found that plaque hemorrhage affects the stresses within atheroma to various degrees depending on age with fresh plaque hemorrhage significantly increasing biomechanical stress.

## Computed tomography

As in previous years, a large number of very high quality, original articles on cardiac CT have been published in 2011. Many of these studies focused on the expanding role of coronary CT angiography (CTA) beyond the detection of lumen stenosis alone. Wang et al. showed the incremental value of dual-energy CT to assess significance of coronary lesions, comparing this technique with quantitative catheter based angiography and SPECT [65, 66]. Other work focused not on intrinsic data derived from coronary luminal contrast opacification [67]. Comparing 2 different contrast injection protocols, Dalager et al. [68] demonstrated the impact of luminal opacification on plaque characterization. Their study with IVUS correlation is evidence that standardized protocols for iodine injection are necessary if plaque characterization is to become more commonly used in clinical practice. In a very important contribution, Lackner and colleagues describe careful assessments of coronary flow, based on computations of CT data in phantoms [69]. Finally, Nagao et al. [70] report that coronary flow information can be derived from variation in coronary enhancement proximal versus distal to a stenotic segment at rest and during adenosine stress. The authors demonstrate that intraluminal coronary iodine density at stress CTA reflects hemodynamic significance of coronary lesions, compared to SPECT and invasive coronary angiography. These findings were apparent even in the presence of inconsistent timing during the bolus passage due to the inherent helical imaging used. This work invites future, precisely controlled studies to determine those conditions for which intraluminal coronary opacification correlates with reference standard hemodynamic significance. Such work is particularly interesting with volume (wide z-coverage) scanners that can acquire the entire coronary tree in a single heart beat.

The journal also published several important epidemiologic papers in different clinical cohorts. Lee et al. [71] reported on the acquisition of CTA data in 806 asymptomatic Chinese subjects, concluding that risk scores could significantly predict the presence of obstructive coronary artery disease. Chan et al. [72] studied 228 Canadian patients and demonstrated that whether or not patients had prior equivocal stress tests, CT imaging identified a substantial fraction of patients with obstructive CAD who required catheterization and subsequent revascularization.

Two important studies focused on the presence of coronary artery disease in patients with a zero calcium score. Ergun et al. [73] reported on 883 patients who underwent CTA after a zero calcium score. In this cohort, over 20 % (180/883) of subjects had noncalcified plaque, and obstructive disease was identified in almost 5 % (n = 43). Clinical risk stratification was determined to be important, as the diabetes and advanced age allowed separating patients with a great likelihood of plaque in the setting of a zero calcium score. Uresky et al. [74] reported on over 1,100 patients with zero coronary calcium detected by CT. While their data confirmed that patients with zero calcium score can have plaque on CTA, contrary to the report of Ergun et al., hemodynamically significant stenosis was rare and clinical factors were not valuable in risk stratification.

Radiation dose remained a hot topic in 2011, and the journal has published several manuscripts related to efforts of exposure reduction [75, 76]. Using prospective ECG gating for coronary CTA with a 64 detector row scanner, Hong et al. [77] observed a mean radiation dose of 3.5 ± 0.3 m Sv. The authors found outstanding test characteristics when coronary artery disease was evaluated on a per-segment basis using catheter angiography as a reference standard. Blankstein et al. [78] report favorable results when comparing image quality of images acquired with 100 versus 120 kV on a dual source CT scanner, with significantly lower radiation dose. The authors conclude that lower kV is preferable for patients who are not obese and therefore would be less likely to have noisy images. Zhang et al. report similar results from a study using 320 × 0.5 mm detector row technology, first introduced in the International Journal of Cardiovascular Imaging, and concluded that both radiation and contrast dose can be reduced by imaging appropriate patients at 100 kV [79–81]. The journal also published articles about iterative reconstruction including a report by Bittencourt et al. [82] that provided initial experience using a 128 × 2 dual source CT scanner. Also, Lee et al. [83] concluded that prospective gating can achieve dose reductions up to 70 % compared to retrospective gating, while maintaining image quality and high diagnostic accuracy in patients undergoing CABG.

The journal has also published a number of interesting meta-analyses. Abdulla and colleagues evaluated 10 coronary CTA studies (over 5,600 subjects) for the purpose of determining the risk of major adverse events [84]. During 21 months follow-up, patients with a normal CTA had an event rate of 0.5 %, and patients with obstructive coronary disease determined by CT had a 16 % event rate; the odds ratio was 6.68 with a 95 % confidence interval of [3.01, 14.8]. Guo et al. [85] followed this report with a meta-analysis focused on the diagnostic accuracy of 32 × 2 detector row dual source scanner that spanned 24 studies. This study was notable for its large scope and the conclusion that this technology has a high sensitivity and negative predictive value. An overview paper was written by Hwang and Choe [86] on the cardiovascular sources of systemic embolism, and its detection and characterization using MSCT and MRI. Sohn et al. [87] concluded in their study that knowledge and awareness of MDCT findings relevant to the Ghent nosology give important clues for the diagnosis of Marfan syndrome that provides better patient care. Finally, Lee et al. [88] concluded that MSCT helps detecting pulmonary embolism in patients after CABG, and that it is encountered more frequently after off-pump CABG than after conventional CABG and in older patients with longer ICU stay.

Beyond coronary imaging, a special issue of the journal focused on imaging in the context of transvascular interventions for valvular and other structural heart disease [35, 36, 89–98]. The articles in the special issue review epidemiological, clinical, and imaging related aspects of these procedures. The central role of image guidance is described for computed tomography, rotational angiography, and echocardiography.
